# Base excess is superior to lactate-levels in prediction of ICU mortality after cardiac surgery

**DOI:** 10.1371/journal.pone.0205309

**Published:** 2018-10-05

**Authors:** Bjoern Zante, Hermann Reichenspurner, Mathias Kubik, Stefan Kluge, Joerg C. Schefold, Carmen A. Pfortmueller

**Affiliations:** 1 Department of Intensive Care Medicine, Inselspital, Bern University Hospital, University of Bern, Bern, Switzerland; 2 Department of Cardiovascular Surgery, University Heart Center Hamburg, Hamburg, Germany; 3 Department of Intensive Care Medicine, Center of Anesthesiology and Intensive Care Medicine, University Medical Center Hamburg-Eppendorf, Hamburg, Germany; IRCCS Policlinico S.Donato, ITALY

## Abstract

**Introduction:**

Cardiac surgery with the use of cardiopulmonary bypass is known to induce distinct metabolic changes. Respective changes in acid-base status including increased systemic lactate levels were previously related to clinical outcomes, but data remain controversial. Therefore, we aim to investigate the relevance of lactate and base excess (BE) levels on ICU-mortality in patients admitted to the ICU after cardiac surgery.

**Materials and methods:**

Perioperative data of patients treated in a tertiary care academic center admitted to the ICU after on-pump surgery were analyzed in a retrospective fashion. Receiver operation characteristic (ROC) curves were constructed for admission lactate-levels and BE with calculation of optimal cut-off values to predict ICU mortality. Univariate followed by multivariate regression models were constructed to identify potential outcome-relevant indices.

**Results:**

Data from 1,058 patients were included in the analysis. Area under the curves for prediction of ICU mortality were 0.79 for lactate levels at ICU admission (sensitivity 61.9%/ specificity 87.5%; optimal cut-off level 3.9mmol/l), and 0.7 for BE (sensitivity 52.4%/ specificity 93.8%, optimal cut-off level -6.7), respectively. Multivariate regression identified BE < -6.7 as the single metabolic predictor of ICU-mortality (HR 4.78, 95%-CI 1.4–16.33, p = 0.01). Explorative subgroup analyses revealed that the combination of lactate ≤3.9mmol/l and BE ≤ -6.7 has stronger impact on mortality than a combination of lactate of >3.9mmol/l and BE > -6.7 (HR 2.56, 95%-CI 0.18–37.17).

**Conclusions:**

At ICU-admission, severely reduced BE appears superior to hyperlactatemia with regard to prediction of ICU-mortality in patients after cardiac surgery.

## Introduction

Cardiac surgery with use of cardiopulmonary bypass (CPB) is known to induce distinct postoperative metabolic changes, which have a major influence on physiological processes [1]. The underlying causes for respective acid-base disturbances after cardiac surgery are manifold and may best be reflected by changes in lactate-levels and base excess (BE).

Elevated lactate levels after cardiac surgery may be caused by anaerobic glycolysis due to tissue hypoxia (known as hyperlactatemia “type A”) related to insufficient macro- or micro-hemodynamics, by pulmonary diseases or by decreased oxygen carrying capacity related to bleeding [2–5]. Likewise, increased lactate levels from non-hypoxic origins are known as “type b”-hyperlactatemia, may occur in cardiosurgical patients [6]. Furthermore, drug therapy [6–8], hypothermia and the usage of CPB [3, 9] may lead to elevated lactate levels post cardiac surgery. Similar like lactate levels, an elevated base deficiency (BD) has many causes post-cardiac surgery [10–12].

Increased lactate levels on ICU were linked to adverse outcomes in general populations of critically ill patients [13–15] and in patients after cardiac surgery [16–19]. In contrast, the influence of base excess and other metabolic indices on ICU mortality remains currently elusive [20–23]. Nevertheless, base excess was shown a strong predictor for mortality in specific subsets of ICU patients such as e.g. major trauma and in patients with cardiogenic shock [24, 25]. However, whether the BD typically seen after cardiac surgery is actually related to the elevated lactate levels is currently controversially discussed [10, 26, 27]. The paucity of data and the controversial discussion on the role of BE on mortality in cardiosurgical patients leads to uncertainty regarding its value as a prognostic tool [11, 28]. We therefore embarked to investigate the prognostic importance of lactate- and BE- levels on ICU mortality after cardiac surgery. Furthermore, we aimed to define lactate- and BE-cut-off values in respect to mortality prediction and to investigate the prognostic role of combinations of metabolic indices in respective critically ill patients.

## Materials and methods

### Setting

Patient data from a 14-bed cardiac surgery ICU of the department of intensive care medicine, University Medical Center Hamburg-Eppendorf during the recording period (February 2009 –March 2010) were analyzed in a retrospective fashion.

### Inclusion/Exclusion criteria

All adult patients (≥18 years) treated at our facility within the study period were eligible for inclusion. Exclusion criteria were as follows: Patients after off-pump surgery, patients who underwent heart-/ and/or lung- transplantation, patients on extracorporeal assist devices, and patients with incomplete data sets.

### Ethical considerations

Ethical approval and informed consent for data analysis and publication of this retrospective, observational, non-interventional investigation using anonymized chart data was not required (Research Ethics Committee of the Hamburg Chamber of Physicians). The analysis was performed in adherence to the Declaration of Helsinki.

### Conduct of the analysis

Preoperative data, EUROscore (European System for Cardiac Operative Risk Evaluation), and preoperative comorbidities such as renal impairment (defined as serum creatinine >200μmol/L), chronic obstructive pulmonary disease (COPD), pulmonary hypertension (PHT, defined as systolic pulmonary artery pressure >60mmHg), severely reduced left ventricular ejection fraction (LVEF, defined as LVEF<35%), re-operation, and emergency operation were recorded.

Standardized treatment was applied for cardio-anesthesia, cardiopulmonary bypass conduction/weaning, and perioperative care in all patients. Standards for conduct of anesthesia, operative procedures, ECC-protocol (pump-flow 2.5l/min/m^2^ body surface, mean arterial pressure 65-50mmHg, mild hypothermia (31–32°C), transfusion trigger: hematocrit <22%) including pump-prime volume, and postoperative care for these patients were unchanged during the study period. Routine point of care blood gas analyses directly after ICU admission were performed from blood samples drawn from arterial catheters inserted in the radial or femoral arteries (Radiometer Copenhagen; ABL 700 Series, Willich, Germany).

Patients extubation criteria were fulfilled when patients were stable on moderate pressure support and PEEP while having a paO_2_>60mmHg and FiO_2_<0.4 as well as the ability to sufficiently protect their airway and follow commands. Patients were discharged from the ICU in case of hemodynamic stabilization without vasoactive medication, stable respiratory conditions, and neurologically adequate.

Complications on the ICU were defined as: surgical revision with/without CPB, cardiopulmonary resuscitation, postoperative coronary angiography for suspected cardiac ischemia, secondary cardiac failure, postoperative respiratory failure (re-intubation, non-invasive ventilation), postoperative infectious complication, delirium, multiple organ failure, acute kidney injury with need for continuous renal replacement therapy (CRRT) and/or others. Moreover, requirement of transfusion of blood products (red packed blood cells, fresh frozen plasma, and/or platelets) within the early postoperative phase (first 12 hours) were noted.

### Statistical analysis

Statistical analysis was performed with MedCalc 17.4 (MedCalc Software, Ostend, Belgium). Normal distribution was tested using the Kolmogorov-Smirnov test. Continuous variables are presented as median and interquartile ranges (IQR). For categorical variables, numbers and proportions are reported, where appropriate.

For lactate- and BE-levels at ICU admission, receiver operating characteristic (ROC) curves and respective areas under the ROC curves (AUC) were constructed. Optimal cut-off values for lactate- and BE-levels were calculated based von ROC-construction and highest sensitivity/specificity to evaluate the diagnostic ability of the respective acid-base parameters. Optimal cut-off levels for lactate and BE were chosen under estimation of an equivalent weighting of sensitivity (Se) and specificity (Sp) according to the following formula: *J* = max_*c*_ [Se (*c*) + Sp (*c*) – 1] (c: cut off-point). Youden´s index was used to define the maximum potential efficiency of lactate- and BE-levels.

Univariate regression analysis followed by multivariate regression were performed to explore the relationship between metabolic parameters and ICU mortality.

A sensitivity analysis including combinations of lactate and BE cut-off values in respect to ICU mortality was performed by Cox proportional hazards regression models. Comparison of lactate-levels in survivors and non-survivors were performed using Chi-square testing. A two-tailed p<0.05 was considered statistical significant.

## Results

### Patient demographics

**Fig 1:**
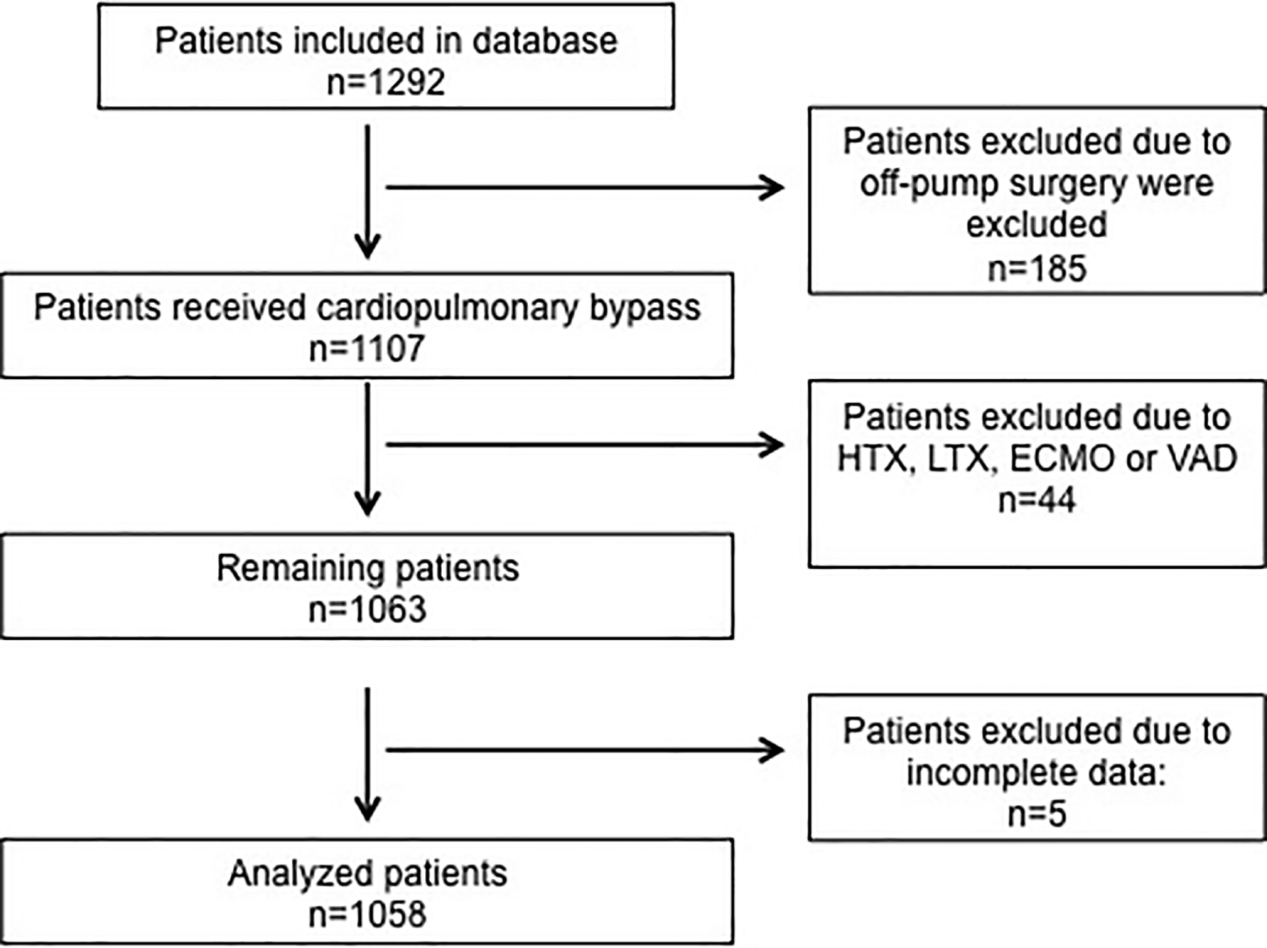
Study flow chart. HTX: heart transplantation, LTX: lung transplantation, ECMO: extracorporeal membrane oxygenation, VAD: ventricular assist device.

Out of 1,292 patients, 1,058 were included in this analysis. The CONSORT flowchart is given in Fig 1. Demographic data are given in Table 1. The median age of the patient population was 69.8 (interquartile range, IQR, 61.1 75.5) with a male predominance (n = 715, 67.6%). General preoperative health status showed a median EUROscore of 4 (IQR 2 6) with less than 10% of all patients suffering from chronic obstructive pulmonary disease (COPD), renal impairment, pulmonary artery hypertension, or severely reduced left ventricular function (Table 1). Seventy-five of all patients (7.1%) underwent an emergency operation. The median duration of cardiopulmonary bypass (CPB)-time was 129 minutes (IQR 103–167 minutes), aortic cross clamp (AOX)–time was 84 minutes (IQR 63–109.8 minutes). At postoperative ICU-admission, median lactate-levels were 1.6mmol/l (IQR 1.0–2.5mmol/l), base excess was -2.5 (IQR -4 to -1.2), respectively. The median duration of mechanical ventilation was 8.7 hours (IQR 5.8–13.0 hours). Twenty-one patients (2%) died on the ICU. In survivors, median lactate-levels were 1.5mmol/l (IQR 1.0–2.4mmol/l), respectively 4.9mmol/l (IQR 4.9–8.9mmol/l) in non-survivors (p = 0.0001). Median base excess was -2.5 (IQR -4 to -1.2) in survivors, and -6.7 (ICR -8.08 to -2.64, p = 0.0007) in non-survivors, respectively.

**Table 1:** Patient demographics and perioperative data.

	all patients
	(n = 1058)
Pre-operative data	
Female gender (%)	343 (32.4)
Age (years)	69.8 (61.1–75.5)
EuroSCORE,	4 (2 to 6)
COPD (%)	85 (8.0)
Renal impairment (%)	64 (6.0)
PHT (%)	30 (2.8)
LVEF <35% (%)	76 (7.2)
Re-operation (%)	40 (3.8)
Emergency operation (%)	75 (7.1)
Intra-operative data	
CPB-Time (min)	129 (103–167)
AOX-Time (min)	84 (63–109.75)
Outcome data	
Length of stay on ICU (days)	2 (2 to 3)
Mechanical ventilation on ICU (hours)	8.7 (5.83–13.0)
Transfusion of blood products on ICU, any type (%)	653 (61.72)
Complications on ICU, any type (%)	99 (9.36)
ICU-mortality (%)	21 (1.98)

Data are presented as median and interquartile ranges, or absolute frequencies. ICU: intensive care unit ICU: intensive care unit; EUROscore: European System for Cardiac Operative Risk Evaluation; COPD: chronic obstructive pulmonary disease; PHT: pulmonary hypertension; LVEF: left ventricular ejection fraction; CPB: cardiopulmonary bypass; AOX: aortic cross clamp.

### Sensitivity and specificity analysis

**Fig 2:**
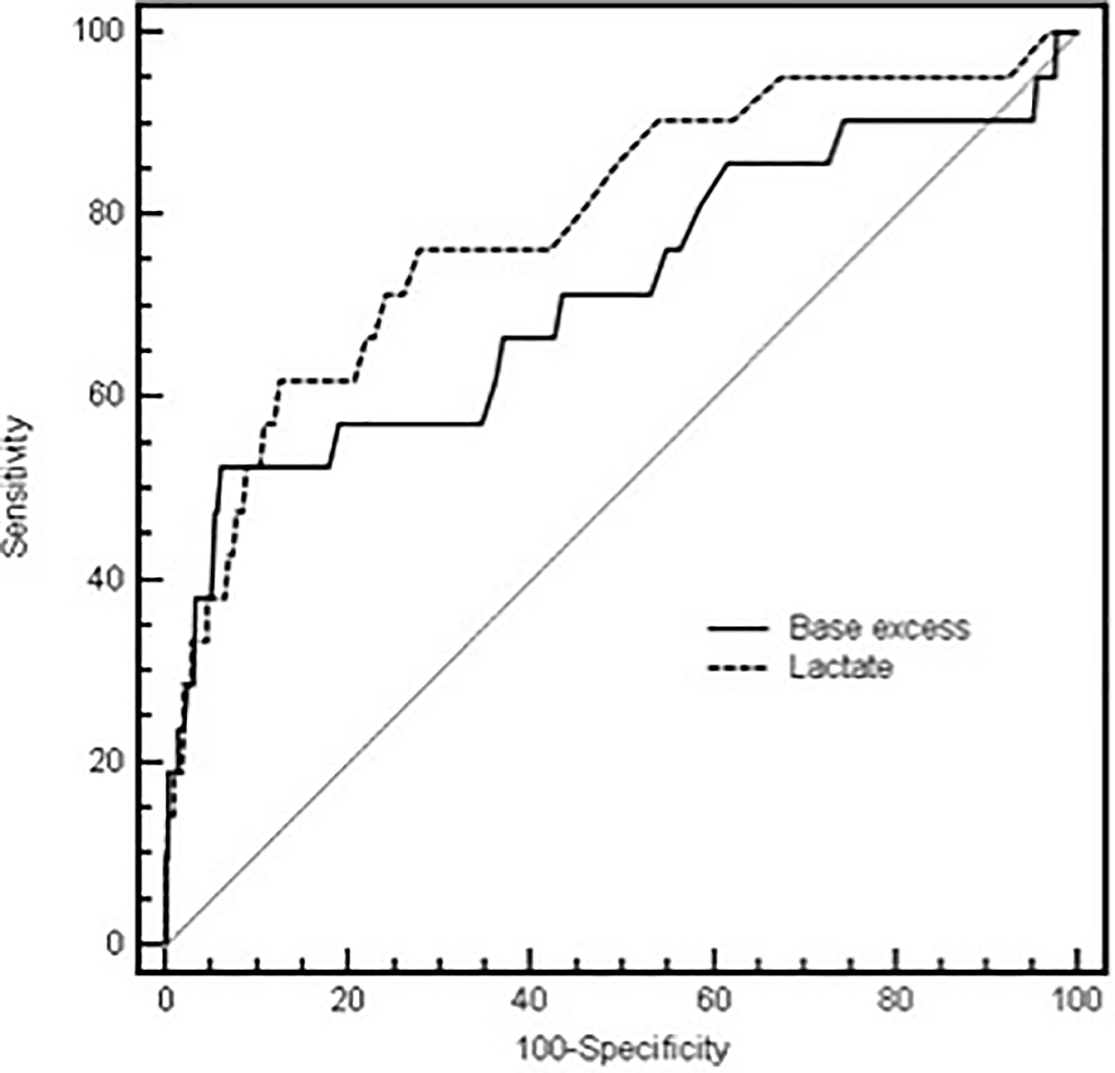
Comparison of receiving operating curves (ROC) for prediction ICU-mortality. Comparison of receiving operating curves (ROC) for prediction of ICU-mortality. Lactate AUROC = 0.79 (95%-CI 0.77–0.82); base excess AUROC = 0.72 (95%-CI 0.69–0.74), p = 0.27.

Receiver operating curves (ROC) for lactate-level and BE at ICU admission were computed in respect to ICU mortality (Fig 2). Pairwise comparison of ROC curves revealed no statistical differences of ROC curves of lactate-level and BE (p = 0.27). The optimal lactate cut-off value to predict ICU mortality was >3.9mmol/l (sensitivity 61.9, specificity 87.5%, Youden´s Index 0.49). For BE the optimal cut-off value to predict ICU mortality was ≤ -6.7 with a sensitivity of 52.4% and a specificity of 93.8%, (Youden´s index 0.46).

### Correlation analysis of lactate levels at ICU admission

Lactate-levels at ICU admission were positively correlated with CPB-time (r = 0.44, p<0.0001, 95%-CI 0.39–0.49), AOX-time (r = 0.32, p<0.0001, 95%-CI 0.27–0.38), and EUROscore (r = 0.27, p<0.0001, 95%-CI 0.21–0.33). A negative correlation was observed for BE (r = -0.48, p<0.0001, 95%-CI -0.52 to -0.43).

### Correlation analysis of base excess at ICU admission

Negative correlation was found for CPB-time (r = -0.12, p = 0.0007, 95%-CI -0.17 to -0.05), AOX-time (r = -0.07, p = 0.03, 95%-CI -0.13 to -0.01), and EUROscore (r = -0.2, p<0.0001, 95%-CI -0.26 to -0.14).

### Univariate and multivariate regression analysis

In univariate regression analysis, EUROscore, CPB-time, pre-existing severe reduced LVEF, re-operation, postoperative complications, lactate-level and BE (continuous values) at ICU admission were related to ICU-mortality (all p<0.05, see Table 2). Furthermore, the lactate-level at ICU admission above the optimal differentiating cut-off value (p = 0.01) and BE (p = 0.0001) below the optimal cut-off value at ICU admission was associated with ICU-mortality. In multivariate Cox regression analysis, BE ≤ -6.7 was revealed as the only independent predictor of ICU-mortality (Hazard ratio 4.78, 95%-CI 1.4–16.33, p = 0.01; overall model fitness Chi^2^ 18.67, p = 0.002). Data on uni- and multivariate analysis are given in Table 2. Despite associations of postoperative complications (any type) with mortality in univariate regression models, inclusion in the multivariate model was rejected in an effort to investigate early potential metabolic indices (at admission) as early predictors. However, in alternative multivariate regression models including postoperative complications (any type) independent predictors of ICU-mortality were CPB-time (HR 1.01, 95%-CI 1.0–1.01, p = 0.04), postoperative complications (HR 8.12, 95%-CI 2.83–23.28, p = 0.0001), and BE ≤ -6.7 at ICU admission (HR 4.41, 95%-CI 1.39–14.03, p = 0.01; overall model fitness Chi^2^ 34.44, p<0.0001).

**Table 2:** Univariate and multivariate Cox proportional hazard regression models for patients variables.

Variables	Univariate model for ICU mortality			Multivariate model for ICU mortality		
	Hazard ratio (95% CI)	*P* value	Wald	Hazard ratio (95% CI)	*P* value	Wald
Preoperative data						
Gender (female)	0.8 (0.31–2.08)	0.64	0.22	-	-	-
Age (per 1 year increase)	1 (0.95–1.03)	0.58	0.3	-	-	-
EUROscore, additive (per 1 step increase)	1.19 (1.05–1.35)	**0.006**	7.65	-	-	-
COPD (y/n)	1.03 (0.24–4.47)	0.97	0.001	-	-	-
Renal impairment (y/n)	0.45 (0.06–3.34)	0.43	0.62	-	-	-
PHT (y/n)	2.86 (0.65–12.51)	0.16	1.94	-	-	-
LVEF <35% (y/n)	3.66 (1.37–9.78)	**0.01**	6.7	2.23 (0.75–6.71)	0.15	2.08
Re-operation (y/n)	3.67 (1.19–11.3)	**0.02**	5.15	3.39 (0.89–12.91)	0.07	3.19
Emergency operation (y/n)	1.47 (0.43–5.03)	0.55	0.37	-	-	-
Intraoperative data						
CPB-Time (per 1 minute increase)	1.01 (1–1.01)	**0.01**	7.63	1 (1–1.01)	0.08	3.05
AOX-Time (per 1 minute increase)	1 (1–1.01)	0.09	2.86	-	-	-
Parameters at ICU admission						
Lactate (per 1mmol/l increase)	1.16 (1.07–1.26)	**0.0003**	13.28	-	**-**	-
Base excess (per one unit increase)	0.83 (0.73–0.93)	**0.002**	9.36	-	-	-
Lactate >3.9mmol/l (y/n)	3.48 (1.33–9.13)	**0.01**	6.44	0.87 (0.25–3.08)	0.83	0.04
Base excess ≤ - 6.7 (y/n)	6.37 (2.5–16.21)	**0.0001**	15.07	4.78 (1.4–16.33)	**0.01**	6.22
Postoperative data						
Mechanical ventilation on ICU (per hours increase)	1 (1–1)	0.87	0.03	-	-	-
Postoperative complications (any type, y/n)	8.74 (3.15–24.27)	<0.001	17.32	-	-	-

Enter Cox-proportional hazards regression. ICU: intensive care unit; EUROscore: European System for Cardiac Operative Risk Evaluation; COPD: chronic obstructive pulmonary disease; PHT: pulmonary hypertension; LVEF: left ventricular ejection fraction; CPB: cardiopulmonary bypass; AOX: aortic cross clamp

### Sensitivity analysis for different combinations of lactate-levels and base excess cut-off values

**Fig 3:**
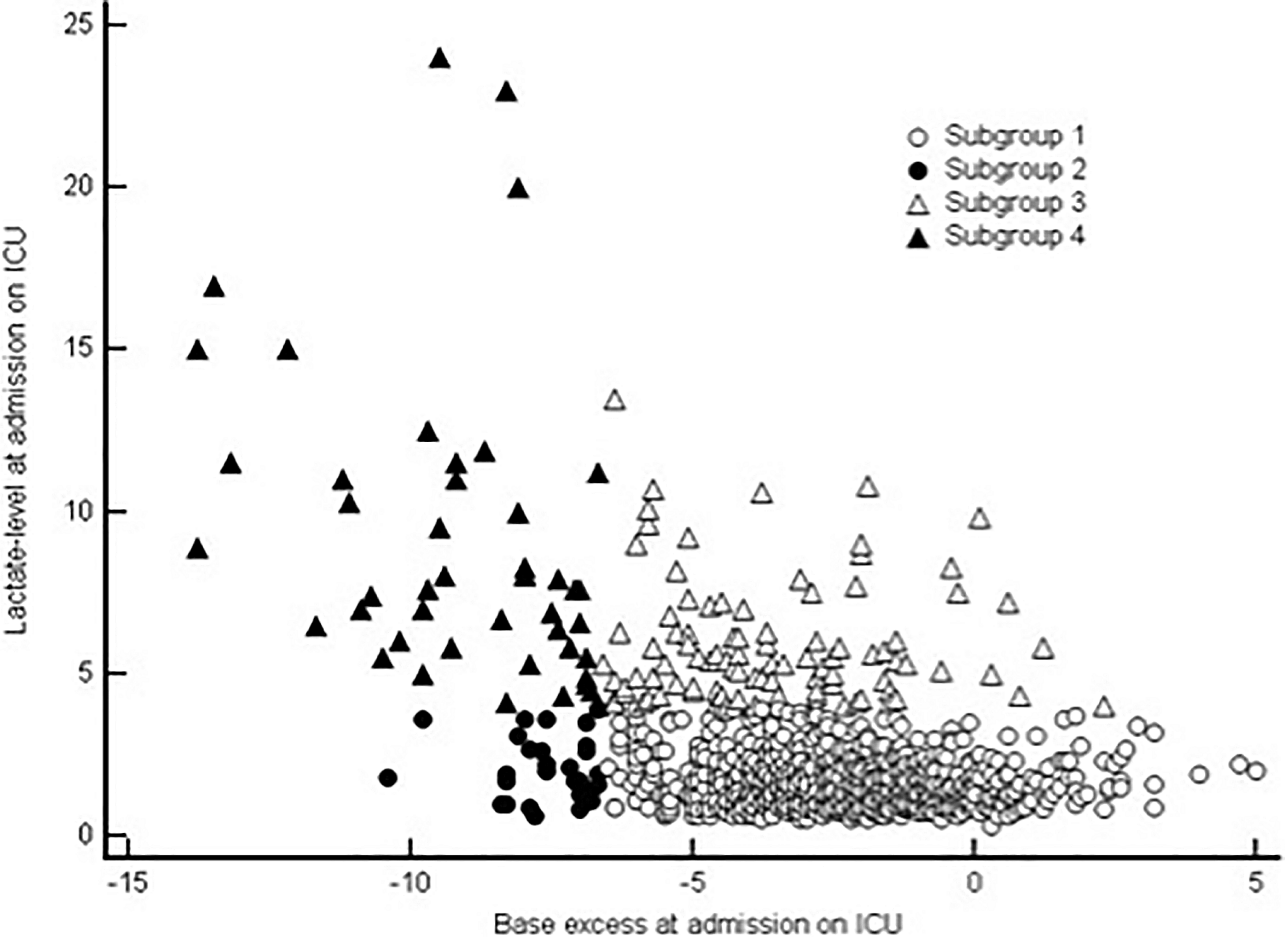
Scatterplot of lactate and base excess levels at admission on ICU after cardiac surgery. 1^st^ group: Lactate ≤3.9mmol/l and BE > -6.7 (n = 887): r = -0.1, p = 0.002, 95%-CI -0.17 to –0.04 2^nd^ group: Lactate ≤3.9mmol/l and BE ≤ -6.7 (n = 28): r = -0.06, p = 0.78, 95%-CI -0.42 to 0.32 3^rd^ group: Lactate >3.9mmol/l and BE > -6.7 (n = 96): r = -0.06, p = 0.58, 95%-CI -0.27 to 0.15. 4^th^ group: Lactate >3.9mmol/l and BE ≤ -6.7 (n = 47): r = -0.36, p = -0.01, 95%-CI -0.59 to -0.08 Overall correlation (n = 1058): r = -0.48, p<0.0001, 95%-CI -0.52 to -0.43.

For further analysis of ICU mortality, patients were divided into four subgroups with respect to their cut-off values for lactate- and BE-levels at ICU admission. A scatterplot of these subgroups is given in Fig 3). The mortality rate for the first subgroup (lactate ≤3.9mmol/l and BE > -6.7) was 0.79% (n = 7/880), for second subgroup (lactate ≤3.9mmol/l and BE ≤ -6.7) 3.7% (n = 1/27), for third subgroup (lactate >3.9mmol/l and BE > -6.7) 3.12% (n = 3/93) and for the fourth subgroup (lactate >3.9mmol/l and BE ≤ -6.7) 21.28% (n = 10/37). Respective hazard ratios and 95%-confidence intervals of the different subgroups are indicated (see Table 3).

**Table 3:** Hazard ratios with 95% confidence intervals and medians for combinations of lactate and base excess.

	1^st^ group Lactate <3.9mmol and BE > -6.7	2^nd^ group Lactate <3.9mmol/ and BE ≤ -6.7	3^rd^ group Lactate >3.9mmol/l and BE > -6.7	4^th^ group Lactate >3.9mmol/l and BE ≤ -6.7
	Hazard ratios with 95% confidence interval			
1^st^ group Lactate <3.9mmol and BE > -6.7	-	2.97 (0.23–39.07)	1.16 (0.4–3.36)	5.76 (1.67–19.94)
2^nd^ group Lactate <3.9mmol/ and BE ≤ -6.7	0.34 (0.02–4.43)	-	0.39 (0.03–5.67)	1.94 (0.12–30.42)
3^rd^ group Lactate >3.9mmol/l and BE > -6.7	0.86 (0.3–2.49)	2.56 (0.18–37.17)	-	4.98 (1.19–20.87)
4^th^ group Lactate >3.9mmol/l and BE ≤ -6.7	0.17 (0.05–0.6)	0.52 (0.03–8.05	0.2 (0.05–0.84)	-

Data for subgroups of lactate and BE cut-off values at admission on ICU

## Discussion

The aim of this analysis was to investigate the relevance of key metabolic indices with regard to prediction of ICU mortality in patients undergoing on-pump cardiac surgery admitted to a tertiary care ICU. Our data show that BE below -6.7 at ICU admission is the only predictor for ICU mortality in a large cohort of mixed cardiosurgical patients.

Base excess as a potential indicator of mortality in post-cardiosurgical patients was scarcely investigated thus far. Few previous studies are limited by lack to assess postoperative mortality [28] or by the fact that multivariate analyses including relevant confounding factors were not performed [11, 17, 28, 29]. In our analysis, we focused on early potential metabolic indices to predict ICU-mortality. We found that severely negative base excess at postoperative ICU admission after cardiosurgery was the only predictor for ICU mortality. This seems important from a clinical perspective and may be relevant for clinicians in efforts for early identification of post-cardiosurgical patients at highest risk. Moreover, in the further course of the ICU-stay, complications increased the risk of ICU-mortality noticeably in addition to severely reduced BE at ICU admission.

In contrast to factors associated with increased lactate levels after cardiac surgery that were extensively studied and mostly reflect circulatory shock states [26, 30, 31], the underlying factors for the systemic reduced BE in patients after cardiac surgery remain less clear. One study linked reduced BE to occult tissue hypo-perfusion [32]. Two further studies in patients with trauma and cardiogenic shock revealed an associated with mortality, but without clear explanations on the underlying pathomechanisms [24, 25]. However, it should be taken into account that decreased BE, as increased lactate, has potential confounders and may be related to multiple etiological factors.

Whether BE or lactate level has better prognostic abilities is controversially discussed so far. Two studies in trauma patients showed that BE is a superior predictor for mortality when compared to lactate [33, 34], while another study in a mixed-surgical ICU cohort showed no difference in mortality predicting properties between lactate and BE [22]. However, multivariate analyses were not performed in these studies.

Only a few studies investigated the relationship between lactate and base excess levels at ICU admission in respect to ICU mortality. In our analysis, the highest mortality was found in the subgroup with hyperlactatemia and severe reductions in base excess (group 4). This appeared not surprising as this constellation may reflect severe lactic acidosis, which was related to a particular high ICU mortality previously [20, 31, 35] and could be related to severe shock states [26, 30, 31]. The second subgroup with a severely reduced BE without concurrent hyperlactatemia has a particular high mortality ratio also. This may point to independent mortality-associated effects of BE.

A common approach for interpretation of biomarkers, especially metabolic indices such as lactate-and BE-levels, is to use the normal ranges provided by hospital laboratories as cut-off values to predict adverse clinical outcomes. However, the utility of these cut-off values for prognostication may highly depend on the sensitivity and specificity of the population in which the biomarker was tested. This is also reflected by significantly different prognostic abilities of lactate levels in trials where an *a priori* laboratory lactate cut-off level of 2mmol/l was used [35–37] when compared to studies where population specific cut-off values were calculated [29]. Our analysis points to the fact that the ideal population based cut-off for lactate for mortality prognostication after cardiac surgery is about 4mmol/l [29]. It was also shown that specificity increased if the cut-off was raised to 5mmol/l but a significant loss of sensitivity must be noted [17]. However, the ideal lactate cut-off for mortality prediction after cardiac surgery is still a matter of on-going debate.

Our analysis has limitations that deserve discussion. First, our study is limited by the relatively small sample size with limited event rates (limited event rates may be considered typical in the specific cohort under investigation). Further, due to the single center design our results may have limited external validity. Moreover, data on long-term follow-up was not available preventing further analyses on mid-and long-term clinical outcomes. Second, and most importantly, we report associations rather than causal relationships. Thus, the underlying reasons for base excess and/or lactate changes remain unclear. Further, we were unable to evaluate patients in regard to liver dysfunction, which could be a reason for increased systemic lactate levels in some patients. Due to the retrospective fashion of this analysis, preoperative baseline characteristics of BE and lactate-levels were not available. Last, neither chloride-levels nor fluid intake, which both impact on the metabolic status of respective patients [8, 15, 23, 38], were recorded, therefore a hyperchloraemic acidosis and/or dilutional acidosis in respective patients cannot be excluded with certainty.

## Conclusions

In a large cohort of post-cardiosurgical patients, we observed that severely reduced base excess at ICU admission is superior to lactate levels for prediction of ICU-mortality. Diminished base excess was the only single independent predictor for ICU mortality after correction for typical confounders, including hyperlactatemia. Subgroup analyses investigating different BE and lactate cut-off levels point towards BE as a key player in respect to mortality prediction in this clinical scenario. Hence, lactate levels interpreted alone as a biomarker provides limited prognostic- and risk-stratification value. Thus, we advocate assessment of admission metabolic conditions after cardiac surgery in an effort to optimize risk stratification in the very early phase of postoperative care on the ICU.
